# Transcriptomic atlas of GNAT family members in pulmonary epithelia under pathological conditions using single‐cell and bulk cell sequencing

**DOI:** 10.1002/ctm2.841

**Published:** 2022-07-20

**Authors:** Xuanqi Liu, Nannan Zheng, Yifei Liu, Bijun Zhu, Jiayun Hou, Mengjia Qian, Linlin Zhang, Liyang Li, Yiming Zeng, Chengshui Chen, Xiangdong Wang

**Affiliations:** ^1^ Department of Pulmonary and Critical Care Medicine, Zhongshan Hospital, Institute for Clinical Science, Shanghai Institute of Clinical Bioinformatics Shanghai Engineering Research for AI Technology for Cardiopulmonary Diseases Shanghai China; ^2^ Jinshan Hospital Centre for Tumor Diagnosis and Therapy Fudan University Shanghai Medical College Shanghai China; ^3^ Center of Molecular Diagnosis and therapy The Second Hospital of Fujian Medical University Quanzhou China; ^4^ Quzhou Hospital of Wenzhou Medical University Quzhou China

Dear Editor,

1

General control non‐repressible 5‐related *N*‐acetyltransferases (GNAT) family members (*n* = 24) are primarily involved in the histone acetylation and translation process, to promote gene expression. Dysfunction of GNAT family genes was found to contribute to cancer cell migration and invasion. Of those, NAT10 and ELP3 were proposed to promote epithelial‐to‐mesenchymal transition, immune cell infiltration and cell circle regulation.^1^ KAT2A, KAT2B, SAT1 and NAA40 were considered as biomarkers of cancer prognosis. NAT1, NAT2, NAT8L, NAA10 and GNPNAT1 were altered in lung cancer, while little has been known about changes of GNAT family members in epithelia of inflammatory lung diseases and in response to challenges. Epithelial cells play important roles in the maintenance of pulmonary defence and mechanic function and act as primary receptors to pathogens and secondary initiators for systemic inflammation.^2^, ^3^ Cancer cells have the capacity of producing chemoattractive factors to recruit the infiltration of immune cells, while immune cell interactions within the microenvironment alter the biological behaviours of cancer cells. The present study aims at understanding transcriptomic phenomes of GNAT family genes in human lung epithelia of lung diseases using single‐cell RNA sequencing (scRNA‐seq), compare GNAT gene heterogeneity of lung cancer with other cancers using bulk RNA‐seq,and exploring GNAT gene changes in normal airway epithelial cells and lung cancer cells in response to external challenges, for example, lipopolysaccharide (LPS), cigarette smoking extract (CSE), cholesterol, lysophosphatidylcholine (LPC) or drugs.

To figure out the role of GNAT in lung epithelial under different pathophysiological conditions including chronic systematic inflammation status, immune system disorders and tumour microenvironment, we analyzed epithelial scRNA‐seq data of 84 lung tissues from 20 healthy controls, 15 patients with chronic obstructive pulmonary disease (COPD), 15 with idiopathic pulmonary fibrosis (IPF), 8 with systemic sclerosis (SSC), 15 with lung adenocarcinoma (LUAD) and 11 corresponding para‐cancer tissues acquired from the databases (GSE128169, GSE131907, GSE136831, GSE128033, E‐MTAB‐6653 and E‐MTAB‐6149).^4^, ^5^, ^6^, ^7^, ^8^ Eight epithelia were labelled and defined, as detailed in Supplemental Materials (Part 1). We found that expression of more GNAT genes (>8) was higher in alveolar type I (Figure 1A) of SSC, alveolar type II of LUAD and SSC (Figure 1B), basal cells of IPF and LUAD (Figure 1C), ciliated epithelia of LUAD (Figure 1D), club epithelia of LUAD and SSC (Figure 1E), goblet cells of LUAD (Figure 1F), mucous epithelia of IPF and LUAD (Figure 1G) and neuroendocrine cells of LUAD (Figure 1H). Of those, KAT2B and NAT10 mainly over‐expressed in COPD, KAT2B, SAT1 and ELP3 in IPF and GNPNAT1, KAT2A, NAA20 and NAT9 in LUAD (*p*‐values in Table S1). mRNA expression of AANAT, NAA11, NAT16, NAT8, NAT8L and SALT1 was hardly detected in most various human lung epithelia or human tumour tissues, of which mean values and SEM were listed in Tables S2 and S3. It seems that the imbalance of histone acetylation and deacetylation plays a critical role in epithelial carcinogenesis and progression of LUAD through altering microtubule stability, chromatin structure, telomerase activity and DNA damage repair.^9^ Some GNAT genes may contribute to the common pathway in the course of LUAD and COPD by mediating the synthesis of CoA‐SH, biological behaviours of epithelia (differentiation, apoptosis and proliferation) and metabolism of amino acids‐related pathways (Figure S1).

**FIGURE 1:**
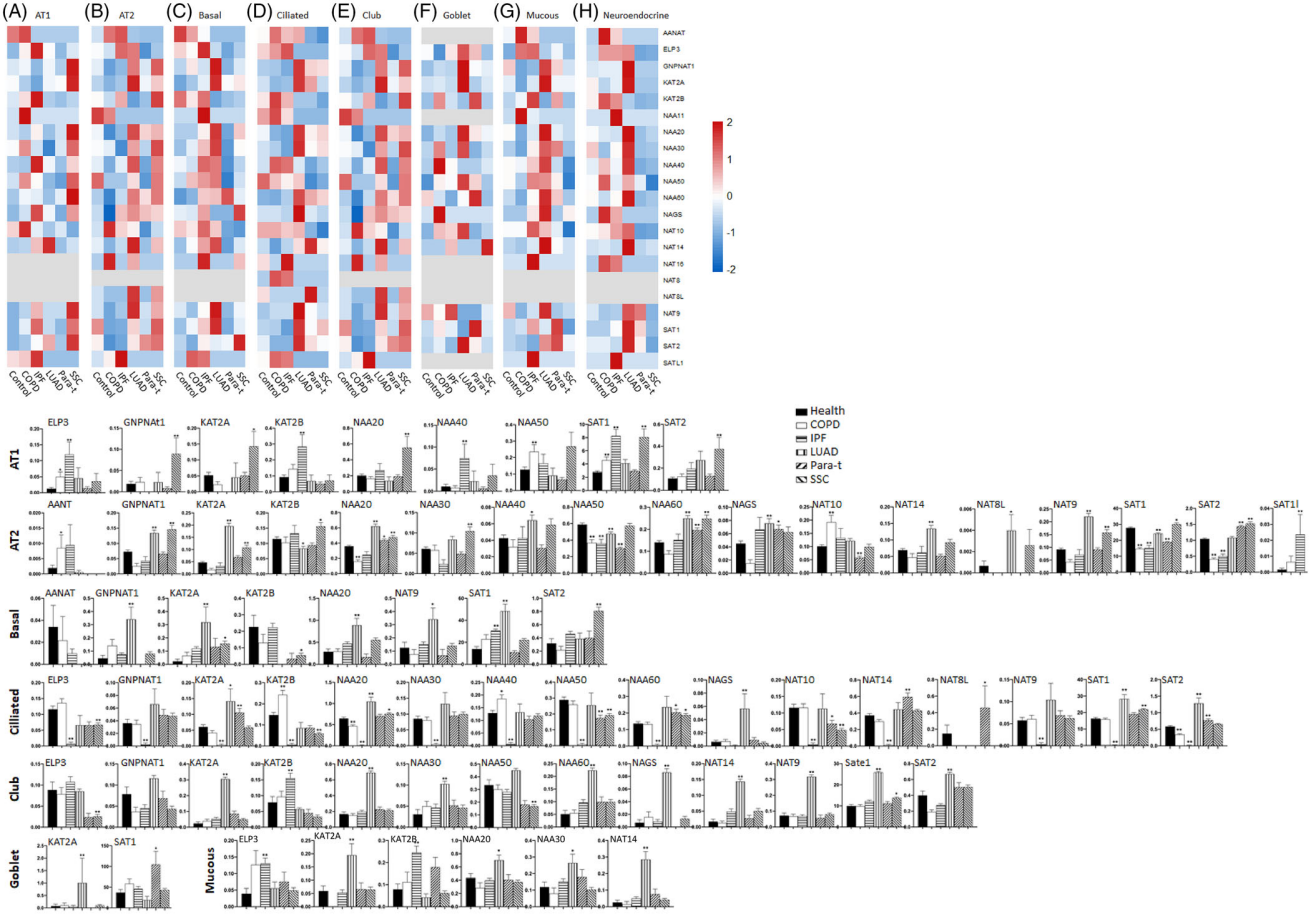
Bar plot shows the expression of the GNAT family gene in 8 kinds of lung epithelial cells including alveolar type I (A), alveolar type II (B), airway basal cells (C), ciliated (D), club (E), goblet (F), mucous (G) and neuroendocrine (H) isolated from five lung samples (lung tissues of healthy controls, chronic obstructive pulmonary disease patients, idiopathic pulmonary fibrosis, lung adenocarcinoma, para‐tumour tissue and systemic sclerosis). * and ** stand for *p*‐values less than 0.05 and 0.01, as compared with healthy controls, respectively

To define the specificity of GNAT gene changes in epithelia of lung cancer, we compared GNAT gene expressions in LUAD with normal lung tissues, para‐cancer tissues and 11 other cancers from the UCSC database (https://xenabroeser.net). The read count value of each GNAT family gene was converted to fragments per kilobase of exon model per million mapped fragments (FPKM) value. Expression folds above the normal of NAT9 were higher in LUAD, NAA20 and NAA50 in LUSC, SAT1 in THCA, NAGS and SAT2 in LIHC, KAT2A in CHOL, NAT8 in KIRP, NAA11 in BLCA or KAT2B, NAT16, NAT8L and SATL1 in GBM (Figure 2A). Expressions of GNAT genes elevated or reduced in various cancers, as compared with normal tissues or LUAD tissues, were presented in Figure 2B and summarized in Table S3. Of those, ELP3, GNPNAT1, NAA20, NAA30, NAA50, NAT10 and NAT14 increased significantly in lung cancers, while AANAT, KAT2A, KAT2B, NAA60, NAT9, SAT1 and SAT2 were lower, of which *p*‐values were shown in Table S3. Different from GNAT genes in LUSC, NAA10, NAA20, NAA40, NAA50 and NAT10 down‐regulated and ELP3, GNPNAT1, KAT2B, NAGS, NAT14 and NAT9 up‐regulated in LUAD tissues (Table S4). The results suggested that GNAT family genes could serve as novel biomarker candidates of epithelial pathologies distinguishing between LUAD and LUSC. Critical GNAT family genes (ELP3, SAT1 and SAT2) may participate in the tumorigenesis of LUAD as well as COPD by regulating chromatin structures and metabolism of polyamines for cellular functions (Figure S1).

**FIGURE 2:**
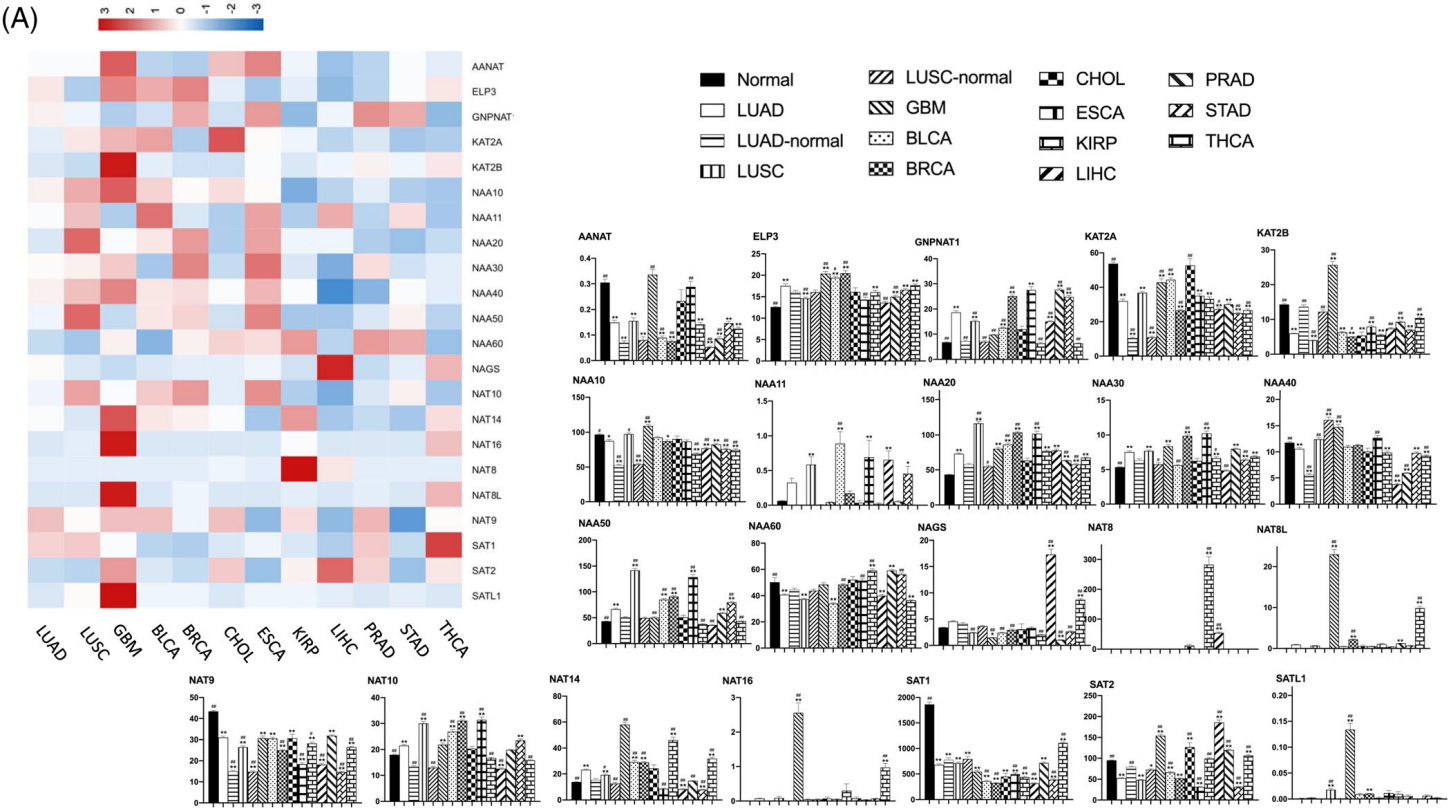
Bar plot of the differential expression of validated GNAT family genes in 15 tissues including normal lung tissue, lung adenocarcinoma (LUAD), para LUAD, LUSC, para‐LUSC, THCA, STAD, PRAD, LIHC, KIRP, ESCA, CHOL, BRCA, BLCA, GBM tissues. * and ** stand for *p*‐values less than 0.05 and 0.01, as compared with healthy controls, respectively. ^#^ and ^##^ stand for 0.05 and 0.01, as compared with LUAD tissue

To uncover responses of GNAT genes to inflammatory stimuli (e.g., CSE, LPS and LysoPC) in normal lung epithelial cells (HBE) and lung cancer cells (A549, SPC‐A1, H1299 and H460), we measured GNAT gene expression in different lung epithelia after exposure of pathogens using bulk RNA‐seq, as detailed in Supplemental Materials.

AANAT and KAT2A were down‐expressed in A549 at 3 h and up‐expressed at 48 h after CSE (Figure 3A,B), implying obvious time‐dependent variations. The molecular mechanisms associated with GNAT expression in A549 challenged with CSE may promote the progression of chronic airway inflammation towards lung cancer, as indicated previously.^10^ NAA20 and NAT10 decreased and SAT1 increased in A549 at 6 h, while LPS induced more GNAT gene changes than CSE at 48 h in A549 and H1299 (Table S5). Cholesterol at 1μg/ml reduced NAT10 expression at 6 h and CSE‐increased KAT2A, NAA20 and NAA40 expression at 24 h (Figure 4A, Table S6). KAT2B and SAT1 upregulated in HBE challenged with CSE for 6 h and showed time‐dependence at 48 h (Figure 4B, Table S6). LysoPC at 100μM reduced the expression of NAA10, NAA20, NAT14 and SAT2 and increased NAA50 in SPC‐A1 cells at 24 h (Figure 4C, Table S7). Effects of LysoPC on SPC‐A1 were mainly focused on the different NAA catalytic submits. AR_2_ inhibitor (No306) reduced the expression of AANAT, KAT2, NAA20, NAA40, NAT14 and NAT8L and increased NAA60, NAT9, SAT1 and SAT2 in A549 cells at 24 h (Figure 4D, Table S7).

**FIGURE 3:**
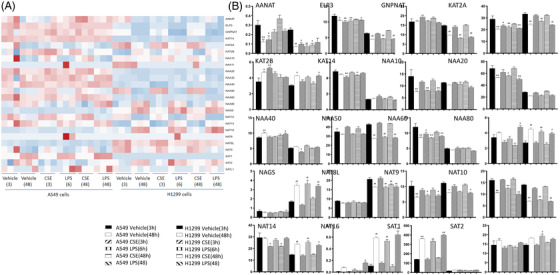
Changes in GNAT family gene expression in response to different challenges. GNAT family gene expression of A549 and H1299 intervened with 6% cigarette smoking extract (CSE) for 3 or 48 h and 1μg/ml LPS for 6 or 48 h as a heatmap (A) and detailed bars (B)

**FIGURE 4:**
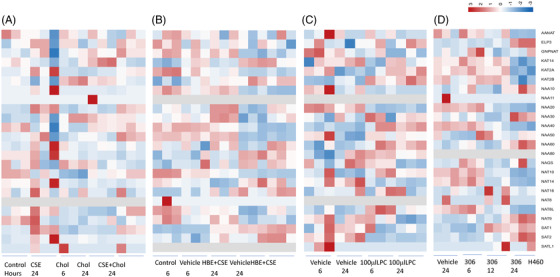
Expressions of GNAT family genes in HBE stimulated with 6% cigarette smoking extract (CSE) for 24 h, 100μM cholesterol for 6 h/24 h, or 6% CSE and 100 μM cholesterol together for 24 h (A) or with 10% CSE for 6 h /24 h (B), in SPC‐A1 cells stimulated with 100 μM lysophosphatidylcholine (LPC) for 6/24h (C) or in H460 cells stimulated with 306‐compound at concentrations of 0.5, 2.5 or 5.0 nM (D)

We also noticed the difference in GNAT gene expression among various epithelial cell lines and among cultured times. Effects of cholesterol and LPC on the GNAT family genes in epithelial cell lines were relatively weak, as compared with CSE and LPS, which implied that the expression of the GNAT family was more prone to change in both chronic inflammation and tumour development. In order to elucidate the potential mechanism of GNAT family genes in lung diseases, we summarized and exampled critical pathways of GNAT family genes‐associated regulations in gene expression of potential targets responsible for the development of lung diseases (Figure S1). It is possible that the dysregulation of GNAT family genes might mediate the dysfunction of lung epithelium through the synthesis of CoA‐SH. We found that the critical gene NAA50 was associated with chromatin organization affecting transcription in COPD which was corresponding to the CSE exposure in normal airway epithelial cells (Figure 4B).

In conclusion, the heterogeneity of transcriptomic profiles exists among GNAT family members, lung epithelia, pulmonary diseases, cancer types, cell‐line types or responses to various challenges. We identified the part of GNAT family genes as specific hallmarks in epithelial cells of lung cancers and the shared features with LUAD and COPD. More obvious expression of GNAT genes was detected in goblet cells of COPD and LUAD, basal and mucous cells of IPF and AT2 and basal cells of SSC. Of those, NAA11, NAT16, NAT8, NAT8L and SATL1 were hardly detected in most human lung epithelia and some showed lung cancer specificificity. The detailed potential pathway and target molecular for GNAT family genes involved in lung epithelium injury of chronic inflammatory diseases need to be furthermore investigated. The current analysis may limit some interpretations. Overall, our data indicate that the heterogeneity and complexity of GNAT family members may contribute to the pathogenesis of lung diseases and can be an important panel for the discovery of lung disease‐specific biomarkers and targets for therapy.

## Supporting information



Supplementary material

Supplementary material

Supplementary material
