# Supplementary material for: Transcriptomic atlas of GNAT family members in pulmonary epithelia under pathological conditions using single‐cell and bulk cell sequencing
Source: Clin Transl Med. 2022 Jul 20;12(7):e841. doi: 10.1002/ctm2.841 (PMC9299758; doi:10.1002/ctm2.841)
Supplement: Supplementary file 1 — Supplementary material [file CTM2-12-e841-s003.docx]

**Supplemental Table 1**. The summary of upregulated or downregulated NAT10 family genes in alveolar type I (AT1) and II (AT2), basal, ciliated, club, Goblet, and mucous epithelia as well as neuroendocrine isolated from lung tissues of patients with COPD, IPF, LUAD, and SSC, as compared with the health (P-value less than 0.05), respectively.

|  |  | Vs COPD | Vs IPF | Vs LUAD | Vs Para-t | Vs SS |
| --- | --- | --- | --- | --- | --- | --- |
| AT1 | Up regulation | ELP3, NAA50 | ELP3, KAT2B, NAA40, NAGS, SAT1 | SAT1, SAT2 | SAT1 | GNPNAT1, KAT2A, NAA20, NAA60, NAT9, SAT1, SAT2 |
|  | Down regulation | —— | —— | —— | KAT2B, NAA50 | —— |
| AT2 | Up regulation | AANAT, NAT10,NAT16, SATL1 | AANAT, SATL1 | ELP3, GNPNAT1, KAT2A, NAA20, NAA40, NAA60, NAGS, NAT10, NAT14, NAT8L, NAT9 | KAT2A, NAA20, NAA60, NAGS, SAT2 | GNPNAT1, KAT2A, KAT2B, NAA20, NAA30, NAA40, NAA60, NAT14,NAGS, NAT9, SAT2 |
|  | Down regulation | GNPNAT1, KAT2A, NAA20, NAA50, NAA60, NAGS, NAT9, SAT1, SAT2 | NAA20, NAA30, NAA50, SAT1, SAT2 | KAT2B, NAA11, NAA50, SAT1 | NAA50, NAT10, NAT14, SAT1 | —— |
| Basal | Up regulation | —— | KAT2A, NAT14 | GNPNAT1, KAT2A, NAA20, NAT14, NAT9, SAT1 | KAT2A, NAA60 | NAA20, NAA30, SAT1, SAT2 |
|  | Down regulation | —— | —— | —— | SAT1 | KAT2B, NAT10 |
| Ciliated | Up regulation | KAT2B, NAA40 | ELP3, NAA40 | GNPNAT1, KAT2A, NAT20, NAGS, SAT1, SAT2 | KAT2A, NAA20, NAA60, NAT14, SAT1, SAT2 | GNPNAT1, NAA20, NAA60, NAT14, SAT1, SAT2 |
|  | Down regulation | KAT2A, NAA20, NAT14, SAT1, SAT2 | NAA20, NAA30, SAT1, SAT2 | —— | ELP3, KAT2B, NAA50, NAT10 | ELP3, KAT2B, NAA50, NAT10 |
| Club | Up regulation | —— | KAT2A, KAT2B, NAA40,NAA60, NAT14, SAT1 | KAT2A, NAA20, NAA30, ,NAA50,NAA60, NAGS, NAT14, NAT9, SAT1, SAT2 | KAT2A, NAA60 | NAA60, NAT14, SAT1, SAT2 |
|  | Down regulation | GNPNAT1, SAT2 | GNPNAT1 | —— | ELP3, NAA50 | ELP3, GNPNAT1, KAT2B, NAA50, NAT10 |
| Goblet | Up regulation | KAT2B, NAT10 | —— | —— | KAT2B | —— |
|  | Down regulation | —— | —— | —— | —— | —— |
| Mucous | Up regulation | ELP3 | ELP3, KAT2B, SAT1 | KAT2A, NAA20, NAA60, NAT14, SAT2 | KAT2B | —— |
|  | Down regulation | KAT2A | GNPNAT1, NAT9 | —— | —— | NAA50, NAT10, SAT1 |
| Neuroendocrine | Up regulation | NAA50, NAT14,SAT1 | —— | GNPNAT1, NAA20, SAT2 | —— | —— |
|  | Down regulation | —— | NAT9 | —— | —— | ELP3, KAT2A, KAT2B, NAA20, NAA40, NAA60, NAT10, NAT9, SAT2 |

**Supplemental Table 2:** Expression of General control non-repressible 5 -related N-acetyltransferases (GNAT) family members in alveolar type I (AT1) and II (AT2), basal, ciliated, club, goblet, and mucous epithelia as well as neuroendocrine cells from patients with chronic obstructive pulmonary disease (COPD), idiopathic pulmonary fibrosis (IPF), systemic sclerosis (SSC), lung adenocarcinoma, and corresponding para- cancer tissues, measured by single cell RNA sequencing (Mean+SEM).

| AT1 | | | | | | | AT2 | | | | | | |
| --- | --- | --- | --- | --- | --- | --- | --- | --- | --- | --- | --- | --- | --- |
|  | Control | COPD | IPF | LUAD | Para-t | SS |  | Control | COPD | IPF | LUAD | Para-t | SS |
| AANAT | 0.006±0.003 | 0.008±0.005 | 0±0 | 0±0 | 0±0 | 0±0 | AANAT | 0.002±0.001 | 0.009±0.004 | 0.009±0.007 | 0.001±0.001 | 0±0 | 0±0 |
| ELP3 | 0.013±0.004 | 0.05±0.015 | 0.119±0.04 | 0.045±0.032 | 0.014±0.005 | 0.036±0.025 | ELP3 | 0.08±0.006 | 0.075±0.014 | 0.1±0.022 | 0.097±0.007 | 0.06±0.006 | 0.07±0.008 |
| GNPNAT1 | 0.019±0.006 | 0.023±0.011 | 0±0 | 0.023±0.023 | 0.008±0.004 | 0.089±0.038 | GNPNAT1 | 0.073±0.005 | 0.026±0.009 | 0.043±0.014 | 0.134±0.01 | 0.066±0.006 | 0.146±0.013 |
| KAT2A | 0.052±0.009 | 0.023±0.009 | 0±0 | 0.045±0.045 | 0.051±0.01 | 0.143±0.047 | KAT2A | 0.047±0.223 | 0.017±0.13 | 0.033±0.18 | 0.196±0.515 | 0.069±0.262 | 0.108±0.337 |
| KAT2B | 0.093±0.013 | 0.143±0.028 | 0.284±0.076 | 0.068±0.038 | 0.051±0.011 | 0.071±0.035 | KAT2B | 0.115±0.007 | 0.103±0.019 | 0.133±0.026 | 0.084±0.007 | 0.095±0.007 | 0.156±0.013 |
| NAA11 | 0±0 | 0.004±0.004 | 0±0 | 0±0 | 0±0 | 0±0 | NAA11 | 0.003±0.001 | 0.002±0.002 | 0±0 | 0±0 | 0±0 | 0±0 |
| NAA20 | 0.203±0.02 | 0.166±0.027 | 0.269±0.084 | 0.136±0.052 | 0.194±0.021 | 0.554±0.142 | NAA20 | 0.359±0.013 | 0.16±0.021 | 0.246±0.043 | 0.621±0.031 | 0.437±0.017 | 0.477±0.024 |
| NAA30 | 0.036±0.009 | 0.062±0.017 | 0.03±0.021 | 0±0 | 0.031±0.008 | 0.071±0.035 | NAA30 | 0.061±0.005 | 0.058±0.012 | 0.024±0.01 | 0.084±0.008 | 0.049±0.005 | 0.105±0.01 |
| NAA40 | 0.011±0.005 | 0.008±0.005 | 0.075±0.032 | 0.023±0.023 | 0.006±0.004 | 0.036±0.025 | NAA40 | 0.043±0.004 | 0.032±0.009 | 0.043±0.014 | 0.064±0.006 | 0.031±0.004 | 0.059±0.007 |
| NAA50 | 0.126±0.015 | 0.236±0.043 | 0.164±0.055 | 0.091±0.044 | 0.067±0.012 | 0.268±0.086 | NAA50 | 0.59±0.018 | 0.368±0.038 | 0.36±0.05 | 0.477±0.025 | 0.301±0.014 | 0.575±0.027 |
| NAA60 | 0.095±0.013 | 0.124±0.024 | 0.119±0.045 | 0.091±0.044 | 0.119±0.016 | 0.232±0.062 | NAA60 | 0.141±0.007 | 0.088±0.015 | 0.152±0.027 | 0.249±0.013 | 0.196±0.011 | 0.248±0.016 |
| NAGS | 0.013±0.005 | 0.019±0.01 | 0.045±0.025 | 0.023±0.023 | 0.01±0.005 | 0.036±0.025 | NAGS | 0.045±0.004 | 0.015±0.006 | 0.066±0.018 | 0.076±0.007 | 0.067±0.006 | 0.062±0.008 |
| NAT10 | 0.038±0.008 | 0.058±0.015 | 0.03±0.021 | 0.023±0.023 | 0.029±0.008 | 0.018±0.018 | NAT10 | 0.101±0.006 | 0.192±0.026 | 0.133±0.036 | 0.122±0.009 | 0.058±0.006 | 0.099±0.01 |
| NAT14 | 0.014±0.005 | 0.015±0.008 | 0.03±0.021 | 0.045±0.032 | 0.012±0.005 | 0.018±0.018 | NAT14 | 0.069±0.005 | 0.047±0.012 | 0.062±0.019 | 0.134±0.01 | 0.051±0.005 | 0.092±0.01 |
| NAT16 | 0±0 | 0±0 | 0±0 | 0±0 | 0±0 | 0±0 | NAT16 | 0±0 | 0.002±0.002 | 0±0 | 0.001±0.001 | 0±0 | 0±0 |
| NAT8 | 0±0 | 0±0 | 0±0 | 0±0 | 0±0 | 0±0 | NAT8 | 0±0 | 0±0 | 0±0 | 0±0 | 0±0 | 0±0 |
| NAT8L | 0±0 | 0±0 | 0±0 | 0±0 | 0±0 | 0±0 | NAT8L | 0.001±0 | 0±0 | 0±0 | 0.004±0.002 | 0±0 | 0.003±0.001 |
| NAT9 | 0.032±0.007 | 0.042±0.013 | 0.075±0.039 | 0.045±0.032 | 0.045±0.009 | 0.107±0.042 | NAT9 | 0.091±0.006 | 0.043±0.009 | 0.071±0.02 | 0.22±0.013 | 0.091±0.007 | 0.157±0.012 |
| SAT1 | 2.776±0.161 | 4.564±0.487 | 8.269±0.965 | 4.091±0.578 | 2.933±0.177 | 8.054±1.269 | SAT1 | 27.81±0.457 | 14.658±0.73 | 15.284±1.517 | 24.159±0.549 | 18.924±0.316 | 30.156±0.75 |
| SAT2 | 0.107±0.013 | 0.127±0.023 | 0.194±0.057 | 0.273±0.082 | 0.131±0.018 | 0.375±0.107 | SAT2 | 1.046±0.024 | 0.408±0.037 | 0.502±0.07 | 1.078±0.04 | 1.436±0.034 | 1.526±0.054 |
| SATL1 | 0.006±0.003 | 0.008±0.005 | 0.015±0.015 | 0±0 | 0±0 | 0±0 | SATL1 | 0.002±0.001 | 0.006±0.004 | 0.024±0.012 | 0±0 | 0±0 | 0±0 |

Green color stands for lower expression of genes with p values less than 0.05, while yellow color for higher expression of gene with p values less than 0.05, as compared with controls, respectively.

| Basal epithelia | | | | | | | Ciliated epithelia | | | | | | |
| --- | --- | --- | --- | --- | --- | --- | --- | --- | --- | --- | --- | --- | --- |
|  | Control | COPD | IPF | LUAD | Para-t | SS |  | Control | COPD | IPF | LUAD | Para-t | SS |
| AANAT | 0.034±0.019 | 0.022±0.022 | 0.01±0.004 | 0±0 | 0±0 | 0±0 | AANAT | 0.007±0.002 | 0.012±0.004 | 0.009±0.001 | 0.009±0.009 | 0.002±0.002 | 0±0 |
| ELP3 | 0.114±0.038 | 0.098±0.035 | 0.19±0.02 | 0.068±0.038 | 0.033±0.033 | 0.059±0.013 | ELP3 | 0.116±0.01 | 0.136±0.014 | 0.16±0.006 | 0.066±0.031 | 0.066±0.011 | 0.067±0.006 |
| GNPNAT1 | 0.045±0.022 | 0.141±0.048 | 0.074±0.012 | 0.341±0.092 | 0±0 | 0.081±0.014 | GNPNAT1 | 0.036±0.006 | 0.035±0.006 | 0.031±0.002 | 0.066±0.024 | 0.049±0.009 | 0.047±0.005 |
| KAT2A | 0.023±0.016 | 0.065±0.026 | 0.119±0.015 | 0.318±0.116 | 0.133±0.063 | 0.156±0.02 | KAT2A | 0.06±0.007 | 0.042±0.007 | 0.047±0.003 | 0.142±0.039 | 0.106±0.014 | 0.059±0.005 |
| KAT2B | 0.227±0.07 | 0.13±0.052 | 0.224±0.025 | 0±0 | 0.033±0.033 | 0.055±0.012 | KAT2B | 0.147±0.012 | 0.243±0.021 | 0.176±0.007 | 0.085±0.027 | 0.086±0.012 | 0.059±0.005 |
| NAA11 | 0±0 | 0±0 | 0.003±0.002 | 0±0 | 0±0 | 0±0 | NAA11 | 0.004±0.002 | 0.006±0.003 | 0.003±0.001 | 0±0 | 0±0 | 0±0 |
| NAA20 | 0.284±0.065 | 0.293±0.057 | 0.472±0.036 | 0.886±0.157 | 0.167±0.069 | 0.557±0.043 | NAA20 | 0.648±0.029 | 0.466±0.028 | 0.436±0.011 | 1.047±0.108 | 0.706±0.042 | 0.757±0.025 |
| NAA30 | 0.057±0.025 | 0.087±0.04 | 0.127±0.017 | 0.182±0.081 | 0.033±0.033 | 0.102±0.015 | NAA30 | 0.085±0.008 | 0.081±0.01 | 0.069±0.004 | 0.132±0.036 | 0.095±0.012 | 0.1±0.007 |
| NAA40 | 0.057±0.025 | 0.043±0.026 | 0.119±0.016 | 0.114±0.048 | 0±0 | 0.038±0.011 | NAA40 | 0.129±0.01 | 0.185±0.016 | 0.18±0.007 | 0.132±0.033 | 0.104±0.013 | 0.119±0.008 |
| NAA50 | 0.341±0.074 | 0.565±0.111 | 0.627±0.045 | 0.682±0.183 | 0.1±0.056 | 0.372±0.035 | NAA50 | 0.288±0.019 | 0.26±0.022 | 0.242±0.008 | 0.255±0.077 | 0.175±0.019 | 0.192±0.01 |
| NAA60 | 0.08±0.033 | 0.098±0.047 | 0.201±0.022 | 0.205±0.07 | 0.267±0.106 | 0.152±0.019 | NAA60 | 0.136±0.012 | 0.133±0.013 | 0.148±0.006 | 0.236±0.065 | 0.204±0.021 | 0.188±0.01 |
| NAGS | 0.011±0.011 | 0.011±0.011 | 0.028±0.007 | 0±0 | 0±0 | 0.04±0.01 | NAGS | 0.007±0.002 | 0.007±0.003 | 0.004±0.001 | 0.057±0.023 | 0.009±0.004 | 0.005±0.002 |
| NAT10 | 0.182±0.057 | 0.163±0.044 | 0.238±0.026 | 0.182±0.067 | 0.067±0.046 | 0.055±0.011 | NAT10 | 0.116±0.01 | 0.116±0.012 | 0.108±0.005 | 0.113±0.045 | 0.067±0.011 | 0.049±0.005 |
| NAT14 | 0.023±0.023 | 0.065±0.03 | 0.114±0.018 | 0.159±0.056 | 0±0 | 0.05±0.011 | NAT14 | 0.372±0.021 | 0.299±0.022 | 0.368±0.01 | 0.443±0.083 | 0.594±0.047 | 0.429±0.017 |
| NAT16 | 0±0 | 0±0 | 0.005±0.003 | 0±0 | 0±0 | 0.002±0.002 | NAT16 | 0.001±0.001 | 0±0 | 0.002±0.001 | 0±0 | 0±0 | 0±0 |
| NAT8 | 0±0 | 0±0 | 0±0 | 0±0 | 0±0 | 0±0 | NAT8 | 0±0 | 0.001±0.001 | 0.001±0 | 0±0 | 0±0 | 0±0 |
| NAT8L | 0±0 | 0±0 | 0±0 | 0±0 | 0±0 | 0±0 | NAT8L | 0.001±0.001 | 0±0 | 0±0 | 0±0 | 0.005±0.003 | 0±0 |
| NAT9 | 0.125±0.042 | 0.076±0.035 | 0.152±0.017 | 0.341±0.086 | 0.067±0.046 | 0.14±0.019 | NAT9 | 0.057±0.007 | 0.061±0.009 | 0.061±0.004 | 0.104±0.038 | 0.069±0.011 | 0.063±0.005 |
| SAT1 | 13.852±2.267 | 22.924±4.066 | 30.482±1.659 | 48.341±6.408 | 10.933±1.505 | 22.123±1.288 | SAT1 | 16.371±0.656 | 15.885±0.688 | 12.795±0.228 | 28.245±2.647 | 19.038±0.99 | 21.958±0.49 |
| SAT2 | 0.318±0.07 | 0.217±0.055 | 0.464±0.035 | 0.386±0.087 | 0.4±0.103 | 0.81±0.055 | SAT2 | 0.586±0.029 | 0.342±0.023 | 0.356±0.009 | 1.283±0.166 | 0.772±0.046 | 0.658±0.02 |
| SATL1 | 0±0 | 0.011±0.011 | 0.01±0.004 | 0±0 | 0±0 | 0±0 | SATL1 | 0.002±0.001 | 0.005±0.003 | 0.005±0.001 | 0±0 | 0±0 | 0±0 |

Green color stands for lower expression of genes with p values less than 0.05, while yellow color for higher expression of gene with p values less than 0.05, as compared with controls, respectively.

| Club epithelia | | | | | | | Goblet cells | | | | | | |
| --- | --- | --- | --- | --- | --- | --- | --- | --- | --- | --- | --- | --- | --- |
|  | Control | COPD | IPF | LUAD | Para-t | SS |  | Control | COPD | IPF | LUAD | Para-t | SS |
| AANAT | 0.003±0.003 | 0.003±0.002 | 0.005±0 | 0±0.003 | 0.003±0 | 0±0 | AANAT | 0±0 | 0±0 | 0±0 | 0±0 | 0±0 | 0±0 |
| ELP3 | 0.089±0.016 | 0.079±0.013 | 0.107±0.006 | 0.085±0.009 | 0.024±0.005 | 0.025±0 | ELP3 | 0.231±0.231 | 0±0 | 0.282±0.09 | 0.5±0.5 | 0.333±0.333 | 0.096±0.041 |
| GNPNAT1 | 0.078±0.011 | 0.037±0.008 | 0.045±0.007 | 0.116±0.017 | 0.068±0.007 | 0.043±0 | GNPNAT1 | 0.077±0.077 | 0.2±0.133 | 0.154±0.059 | 1±0 | 0.333±0.333 | 0.269±0.078 |
| KAT2A | 0.024±0.011 | 0.039±0.009 | 0.054±0.013 | 0.304±0.016 | 0.086±0.007 | 0.049±0 | KAT2A | 0.077±0.077 | 0.1±0.1 | 0.051±0.051 | 1±1 | 0±0 | 0.096±0.041 |
| KAT2B | 0.078±0.017 | 0.097±0.016 | 0.155±0.005 | 0.057±0.012 | 0.045±0.006 | 0.033±0 | KAT2B | 0±0 | 0.5±0.167 | 0.128±0.054 | 0±0 | 0.667±0.333 | 0.058±0.033 |
| NAA11 | 0±0 | 0±0 | 0±0 | 0±0 | 0±0 | 0±0 | NAA11 | 0±0 | 0±0 | 0±0 | 0±0 | 0±0 | 0±0 |
| NAA20 | 0.167±0.022 | 0.149±0.017 | 0.197±0.021 | 0.691±0.031 | 0.226±0.016 | 0.212±0 | NAA20 | 0.308±0.133 | 0.6±0.306 | 0.436±0.115 | 2±0 | 1±1 | 0.808±0.14 |
| NAA30 | 0.031±0.012 | 0.05±0.008 | 0.046±0.007 | 0.103±0.013 | 0.051±0.007 | 0.046±0 | NAA30 | 0.077±0.077 | 0.4±0.4 | 0.051±0.036 | 0.5±0.5 | 0±0 | 0.096±0.05 |
| NAA40 | 0.024±0.009 | 0.034±0.009 | 0.057±0.005 | 0.06±0.005 | 0.007±0.005 | 0.024±0 | NAA40 | 0±0 | 0.3±0.153 | 0.077±0.057 | 0±0 | 0±0 | 0.038±0.027 |
| NAA50 | 0.334±0.036 | 0.301±0.021 | 0.28±0.015 | 0.45±0.031 | 0.182±0.014 | 0.167±0 | NAA50 | 0.308±0.133 | 0.9±0.348 | 0.821±0.168 | 1.5±1.5 | 1±0.577 | 0.558±0.118 |
| NAA60 | 0.051±0.012 | 0.055±0.012 | 0.096±0.01 | 0.223±0.02 | 0.099±0.01 | 0.099±0 | NAA60 | 0.615±0.213 | 0.4±0.163 | 0.205±0.075 | 0.5±0.5 | 1±0 | 0.288±0.079 |
| NAGS | 0.007±0.008 | 0.016±0.003 | 0.009±0.006 | 0.086±0 | 0±0.004 | 0.013±0 | NAGS | 0±0 | 0.1±0.1 | 0±0 | 0±0 | 0±0 | 0±0 |
| NAT10 | 0.089±0.025 | 0.154±0.013 | 0.114±0.007 | 0.127±0.013 | 0.048±0.007 | 0.043±0 | NAT10 | 0±0 | 0.3±0.153 | 0.256±0.088 | 0.5±0.5 | 0±0 | 0.077±0.06 |
| NAT14 | 0.017±0.006 | 0.013±0.008 | 0.049±0.008 | 0.144±0.01 | 0.027±0.008 | 0.051±0 | NAT14 | 0±0 | 0±0 | 0.026±0.026 | 0±0 | 0±0 | 0.058±0.033 |
| NAT16 | 0±0 | 0±0.001 | 0.001±0.001 | 0.003±0 | 0±0 | 0±0 | NAT16 | 0±0 | 0±0 | 0±0 | 0±0 | 0±0 | 0±0 |
| NAT8 | 0±0 | 0±0 | 0±0 | 0±0 | 0±0 | 0±0 | NAT8 | 0±0 | 0±0 | 0±0 | 0±0 | 0±0 | 0±0 |
| NAT8L | 0±0 | 0±0 | 0±0.002 | 0.01±0 | 0±0 | 0±0 | NAT8L | 0±0 | 0±0 | 0±0 | 0±0 | 0±0 | 0±0 |
| NAT9 | 0.072±0.014 | 0.068±0.009 | 0.068±0.012 | 0.318±0.017 | 0.058±0.009 | 0.08±0 | NAT9 | 0.154±0.104 | 0.1±0.1 | 0.231±0.093 | 0±0 | 0±0 | 0.077±0.037 |
| SAT1 | 9.918±0.728 | 9.901±0.496 | 12.238±0.427 | 25.896±0.82 | 11.182±0.475 | 13.8±0 | SAT1 | 37.154±7.276 | 58.3±11.798 | 46.256±5.717 | 19±9 | 104.667±31.84 | 42.904±3.789 |
| SAT2 | 0.399±0.026 | 0.191±0.02 | 0.275±0.024 | 0.669±0.056 | 0.503±0.026 | 0.505±0 | SAT2 | 0.538±0.215 | 0.6±0.221 | 0.487±0.109 | 1±1 | 0.667±0.333 | 0.558±0.111 |
| SATL1 | 0.003±0.004 | 0.005±0.003 | 0.006±0 | 0±0 | 0±0 | 0±0 | SATL1 | 0±0 | 0±0 | 0±0 | 0±0 | 0±0 | 0±0 |

Green color stands for lower expression of genes with p values less than 0.05, while yellow color for higher expression of gene with p values less than 0.05, as compared with controls, respectively.

| Mucous epithelia | | | | | | | Neuroendocrine cells | | | | | | |
| --- | --- | --- | --- | --- | --- | --- | --- | --- | --- | --- | --- | --- | --- |
|  | Control | COPD | IPF | LUAD | Para-t | SS |  | Control | COPD | IPF | LUAD | Para-t | SS |
| AANAT | 0.007±0.007 | 0.032±0.022 | 0.013±0.005 | 0±0 | 0±0 | 0±0 | AANAT | 0±0 | 0.034±0.034 | 0.012±0.009 | 0±0 | 0±0 | 0±0 |
| ELP3 | 0.039±0.016 | 0.127±0.042 | 0.131±0.016 | 0.056±0.019 | 0.075±0.029 | 0.049±0.008 | ELP3 | 0.019±0.014 | 0.057±0.03 | 0.055±0.018 | 0.059±0.033 | 0±0 | 0.002±0.002 |
| GNPNAT1 | 0.158±0.035 | 0.063±0.031 | 0.079±0.014 | 0.222±0.037 | 0.151±0.042 | 0.106±0.013 | GNPNAT1 | 0.01±0.01 | 0.011±0.011 | 0.012±0.009 | 0.078±0.038 | 0±0 | 0±0 |
| KAT2A | 0.059±0.019 | 0±0 | 0.054±0.01 | 0.194±0.043 | 0.066±0.024 | 0.065±0.01 | KAT2A | 0.048±0.021 | 0.011±0.011 | 0.031±0.014 | 0.157±0.059 | 0±0 | 0±0 |
| KAT2B | 0.079±0.024 | 0.111±0.046 | 0.245±0.029 | 0.042±0.017 | 0.179±0.044 | 0.062±0.01 | KAT2B | 0.067±0.034 | 0.159±0.088 | 0.123±0.047 | 0.02±0.02 | 0±0 | 0.002±0.002 |
| NAA11 | 0±0 | 0.016±0.016 | 0.002±0.002 | 0±0 | 0±0 | 0±0 | NAA11 | 0±0 | 0±0 | 0.006±0.006 | 0±0 | 0±0 | 0±0 |
| NAA20 | 0.434±0.065 | 0.286±0.076 | 0.395±0.034 | 0.701±0.079 | 0.406±0.079 | 0.374±0.026 | NAA20 | 0.154±0.043 | 0.102±0.036 | 0.092±0.029 | 0.588±0.119 | 0±0 | 0.055±0.012 |
| NAA30 | 0.092±0.024 | 0.079±0.041 | 0.109±0.014 | 0.139±0.029 | 0.132±0.038 | 0.086±0.011 | NAA30 | 0.029±0.021 | 0±0 | 0.018±0.011 | 0.059±0.033 | 0±0 | 0.002±0.002 |
| NAA40 | 0.066±0.02 | 0.063±0.031 | 0.074±0.012 | 0.097±0.028 | 0.057±0.023 | 0.037±0.007 | NAA40 | 0.029±0.016 | 0.057±0.025 | 0.031±0.014 | 0.098±0.051 | 0±0 | 0.005±0.003 |
| NAA50 | 0.447±0.061 | 0.381±0.094 | 0.535±0.042 | 0.528±0.071 | 0.453±0.082 | 0.296±0.022 | NAA50 | 0.192±0.094 | 0.341±0.084 | 0.172±0.039 | 0.275±0.093 | 0.167±0.167 | 0.014±0.007 |
| NAA60 | 0.118±0.031 | 0.079±0.034 | 0.151±0.017 | 0.264±0.053 | 0.179±0.046 | 0.104±0.012 | NAA60 | 0.048±0.021 | 0.023±0.016 | 0.049±0.019 | 0.157±0.059 | 0±0 | 0.01±0.005 |
| NAGS | 0±0 | 0±0 | 0.007±0.004 | 0.021±0.012 | 0±0 | 0.007±0.003 | NAGS | 0±0 | 0.011±0.011 | 0.006±0.006 | 0±0 | 0±0 | 0±0 |
| NAT10 | 0.112±0.03 | 0.111±0.051 | 0.177±0.02 | 0.139±0.031 | 0.104±0.03 | 0.029±0.006 | NAT10 | 0.048±0.029 | 0.057±0.025 | 0.08±0.026 | 0.098±0.051 | 0±0 | 0.002±0.002 |
| NAT14 | 0.026±0.013 | 0.016±0.016 | 0.05±0.01 | 0.285±0.049 | 0.075±0.032 | 0.042±0.008 | NAT14 | 0.019±0.014 | 0.023±0.016 | 0.006±0.006 | 0.255±0.111 | 0±0 | 0.019±0.007 |
| NAT16 | 0±0 | 0±0 | 0.004±0.003 | 0±0 | 0±0 | 0±0 | NAT16 | 0±0 | 0.023±0.023 | 0.018±0.011 | 0±0 | 0±0 | 0±0 |
| NAT8 | 0±0 | 0±0 | 0±0 | 0±0 | 0±0 | 0±0 | NAT8 | 0±0 | 0±0 | 0±0 | 0±0 | 0±0 | 0±0 |
| NAT8L | 0±0 | 0±0 | 0±0 | 0±0 | 0±0 | 0±0 | NAT8L | 0±0 | 0±0 | 0±0 | 0±0 | 0±0 | 0±0 |
| NAT9 | 0.164±0.032 | 0.079±0.034 | 0.113±0.016 | 0.222±0.04 | 0.094±0.032 | 0.108±0.013 | NAT9 | 0.058±0.023 | 0.023±0.016 | 0.031±0.014 | 0.196±0.056 | 0.167±0.167 | 0±0 |
| SAT1 | 29.572±3.394 | 30.095±3.002 | 35.251±1.672 | 30.639±2.627 | 38.538±3.53 | 26.724±0.982 | SAT1 | 2.538±0.301 | 2.58±0.408 | 4.908±0.608 | 12.804±2.38 | 6.833±2.372 | 1.561±0.09 |
| SAT2 | 0.428±0.062 | 0.413±0.092 | 0.389±0.031 | 0.563±0.069 | 0.613±0.08 | 0.466±0.029 | SAT2 | 0.135±0.041 | 0.136±0.046 | 0.104±0.027 | 0.353±0.073 | 0.167±0.167 | 0.038±0.01 |
| SATL1 | 0±0 | 0±0 | 0.002±0.002 | 0±0 | 0±0 | 0±0 | SATL1 | 0±0 | 0±0 | 0.006±0.006 | 0±0 | 0±0 | 0±0 |

Green color stands for lower expression of genes with p values less than 0.05, while yellow color for higher expression of gene with p values less than 0.05, as compared with controls, respectively.

**Supplemental Table 3:** Expression of general control non-repressible 5 -related N-acetyltransferases (GNAT) family members in various tumor tissues and corresponding para-cancer tissues, measured by bulk RNA sequencing (Mean+SEM).

|  | Normal | LUAD | LUAD-P | LUSC | LUSC-P | GBM | BLCA | BRCA | CHOL | ESCA | KIRP | LIHC | PRAD | STAD | THCA |
| --- | --- | --- | --- | --- | --- | --- | --- | --- | --- | --- | --- | --- | --- | --- | --- |
| AANAT | 0.305±0.013 | 0.149±0.009 | 0.069±0.008 | 0.155±0.012 | 0.08±0.013 | 0.336±0.022 | 0.09±0.007 | 0.075±0.005 | 0.233±0.045 | 0.288±0.021 | 0.141±0.008 | 0.053±0.003 | 0.086±0.006 | 0.146±0.008 | 0.125±0.006 |
| ELP3 | 12.61±0.164 | 17.48±0.549 | 16.03±0.436 | 14.64±0.274 | 16.03±0.496 | 20.36±0.537 | 19.4±0.293 | 20.45±0.596 | 16.12±0.986 | 14.32±0.581 | 16.02±0.404 | 13.61±0.276 | 15.02±0.325 | 16.58±0.306 | 17.66±0.305 |
| GNPNAT1 | 6.742±0.133 | 18.64±0.693 | 7.17±0.318 | 15.18±0.372 | 6.946±0.37 | 9.766±0.296 | 12.51±0.319 | 25.04±0.526 | 11.94±1.215 | 27.54±1.344 | 6.412±0.267 | 15.06±0.436 | 27.65±0.617 | 24.76±0.659 | 6.42±0.138 |
| KAT2A | 53.72±1.074 | 32.09±1.185 | 10.48±0.55 | 36.73±0.921 | 10.94±0.729 | 42.76±2.222 | 44.33±1.05 | 26.59±0.463 | 52.72±3.899 | 35.08±1.102 | 33.27±1.301 | 27.35±0.693 | 30.13±0.671 | 24.9±0.654 | 26.63±0.716 |
| KAT2B | 14.33±0.228 | 5.955±0.153 | 13.66±0.534 | 4.009±0.101 | 12.21±0.547 | 25.65±1.022 | 6.313±0.259 | 4.981±0.101 | 5.334±1.08 | 7.93±0.408 | 5.573±0.207 | 7.365±0.289 | 9.288±0.224 | 6.998±0.222 | 10.41±0.276 |
| NAA10 | 96.81±1.132 | 87.63±2.097 | 52.76±1.249 | 97.32±1.77 | 53.97±1.477 | 109±2.066 | 92.61±1.553 | 87.31±1.995 | 90.47±4.104 | 86.96±2.439 | 69.38±1.333 | 77.01±1.376 | 82.66±1.497 | 75.99±1.459 | 74.85±1.425 |
| NAA11 | 0.063±0.005 | 0.324±0.064 | 0.003±0.001 | 0.586±0.116 | 0.006±0.002 | 0.043±0.006 | 0.891±0.168 | 0.172±0.038 | 0.034±0.034 | 0.693±0.242 | 0.02±0.013 | 0.655±0.128 | 0.051±0.016 | 0.453±0.109 | 0.001±0 |
| NAA20 | 43.18±0.514 | 72.89±1.422 | 57.86±1.198 | 116.6±2.599 | 54.64±1.838 | 80.2±2.087 | 86.04±1.967 | 103.7±1.448 | 63.29±3.481 | 101.6±3.87 | 76.51±1.528 | 77.95±1.834 | 63.39±0.757 | 57.87±1.26 | 67.5±0.789 |
| NAA30 | 5.345±0.061 | 7.536±0.199 | 6.347±0.201 | 7.692±0.146 | 5.744±0.246 | 8.373±0.253 | 5.59±0.129 | 9.855±0.14 | 6.246±0.391 | 10.23±0.322 | 6.642±0.19 | 4.862±0.12 | 8.007±0.13 | 6.409±0.148 | 6.912±0.113 |
| NAA40 | 11.78±0.154 | 10.58±0.241 | 5.589±0.244 | 12.42±0.271 | 16.08±0.507 | 14.72±0.511 | 10.98±0.278 | 11.3±0.158 | 10.07±0.672 | 12.7±0.406 | 9.879±0.276 | 3.794±0.158 | 5.959±0.105 | 9.783±0.232 | 9.256±0.168 |
| NAA50 | 43.07±0.538 | 66.47±1.615 | 50.52±1.526 | 141.7±3.058 | 49.73±1.865 | 50.29±1.452 | 84.84±2.19 | 90.93±1.47 | 50.94±3.692 | 128.5±5.377 | 37.52±1.446 | 35.92±1.059 | 58.58±1.256 | 79.55±1.89 | 43.39±0.81 |
| NAA60 | 50.22±3.365 | 40.76±0.642 | 44.54±1.189 | 37.34±0.647 | 43.78±1.306 | 48.57±1.214 | 34.04±0.574 | 48.6±0.54 | 52.1±2.332 | 51.41±1.303 | 59.15±1.147 | 39.98±0.855 | 59.13±0.693 | 56.12±1.028 | 36.46±0.466 |
| NAGS | 3.37±0.095 | 4.546±0.16 | 4.101±0.216 | 2.425±0.086 | 3.619±0.171 | 1.467±0.051 | 2.41±0.104 | 2.948±0.139 | 3.085±1.008 | 3.276±0.22 | 2.029±0.118 | 17.29±1.058 | 1.036±0.033 | 2.649±0.137 | 8.67±0.239 |
| NAT10 | 17.95±0.265 | 21.53±0.374 | 13.32±0.35 | 30.01±0.747 | 13.02±0.529 | 21.87±0.603 | 26.82±0.807 | 31.04±0.455 | 20.31±0.915 | 31.41±1.125 | 16.67±0.29 | 12.59±0.297 | 19.82±0.198 | 23.53±0.533 | 15.84±0.146 |
| NAT14 | 13.8±0.28 | 23.2±0.623 | 15.41±0.985 | 19.16±0.605 | 12.73±0.74 | 57.96±1.823 | 29.18±1.253 | 28.86±0.624 | 24.21±2.843 | 8.692±0.496 | 46.12±2.205 | 8.336±0.53 | 14.54±0.376 | 7.975±0.315 | 31.76±1.093 |
| NAT16 | 0.005±0.001 | 0.075±0.011 | 0.007±0.004 | 0.089±0.041 | 0.002±0.001 | 2.562±0.285 | 0.048±0.013 | 0.05±0.013 | 0.07±0.044 | 0.052±0.012 | 0.299±0.196 | 0.078±0.036 | 0.035±0.003 | 0.068±0.009 | 0.97±0.119 |
| NAT8 | 0.272±0.013 | 0.079±0.01 | 0.148±0.021 | 0.125±0.021 | 0.148±0.021 | 0.092±0.009 | 0.28±0.038 | 0.334±0.02 | 11.89±3.092 | 0.237±0.053 | 282.8±26.37 | 54.59±2.941 | 0.128±0.009 | 0.523±0.072 | 0.095±0.012 |
| NAT8L | 0.121±0.004 | 0.936±0.098 | 0.086±0.007 | 0.686±0.058 | 0.083±0.009 | 23.01±1.237 | 0.497±0.06 | 2.22±0.085 | 0.57±0.227 | 0.516±0.086 | 1.062±0.22 | 0.405±0.091 | 1.257±0.052 | 0.688±0.114 | 9.857±0.436 |
| NAT9 | 43.4±0.488 | 30.97±0.623 | 14.98±0.439 | 26.46±0.547 | 14.77±0.551 | 30.7±0.981 | 30.47±0.622 | 24.98±0.354 | 30.62±1.856 | 18.4±0.632 | 28.23±0.71 | 18.19±0.479 | 31.8±0.374 | 14.65±0.332 | 26.48±0.346 |
| SAT1 | 1863±48.17 | 680±17.14 | 775±40.82 | 710±21.23 | 793.5±50.76 | 543.2±27.97 | 359.5±14.27 | 324.7±6.622 | 447±67.34 | 495.7±25.39 | 436.9±14.74 | 316.8±9.408 | 714.4±21.24 | 389.2±13.25 | 1108±19.14 |
| SAT2 | 94.84±0.83 | 52.84±1.072 | 79.02±1.819 | 48.67±1.037 | 72.34±2.304 | 154±4.293 | 66.15±1.79 | 51.78±0.747 | 126.5±10.87 | 31.64±1.26 | 99.09±2.804 | 185.8±6.256 | 119.7±2.105 | 31.82±0.672 | 106.3±2.146 |
| SATL1 | 0.001±0.0003 | 0.002±0.0004 | 0.001±0.0004 | 0.018±0.003 | 0.001±0.0004 | 0.134±0.0123 | 0.009±0.0021 | 0.01±0.001 | 0.002±0.0013 | 0.012±0.0045 | 0.01±0.0046 | 0.006±0.0019 | 0.001±0.0002 | 0.006±0.0014 | 0.002±0.0003 |

Green color stands for lower expression of genes with p values less than 0.05, while yellow color for higher expression of gene with p values less than 0.05, as compared with controls, respectively.

**Supplemental Table 4**. The summary of upregulated or downregulated NAT10 family genes in lung tissues of patients with LUSC or LUAD, as compared with other 11 type cancers (P-value less than 0.05), respectively.

|  |  | LUSC | LUAD | THCA | STAD | PRAD | LIHC | KIRP | ESCA | CHOL | BRCA | BLCA | GBM |
| --- | --- | --- | --- | --- | --- | --- | --- | --- | --- | --- | --- | --- | --- |
| LUSC | Up regulation | ——— | ELP3, GNPNAT1, KAT2B, NAGS, NAT14, NAT9 | ELP3, KAT2B, NAGS, NAT14, NAT16, NAT8L, SAT1, SAT2 | GNPNAT1, ELP3, KAT2B, NAA60 | GNPNAT1, KAT2B, NAA60, NAT9, SAT2 | KAT2B, NAGS, NAT8, SAT2 | NAA60, NAT14, NAT8, SAT2 | AANAT, GNPNAT1, KAT2B, NAA30, NAA60 | KAT2A, SAT2 | ELP3,GNPNAT1,KAT2B,NAA30,NAA60,NAT14,NAT8L | ELP3,KAT2A,KAT2B,NAT14,NAT9,SAT2 | AANAT,ELP3,KAT2A,KAT2B,NAA10,NAA40,NAA60,NAT14,NAT16,NAT8L,NAT9,SAT2 |
|  | Down regulation | ——— | NAA10,NAA20,NAA40,NAA50,NAT10 | GNPNAT1,KAT2A,NAA10,NAA11,NAA20,NAA30,NAA40,NAA50,NAT10 | KAT2A,NAA10,NAA20,NAA30,NAA40,NAA50,NAT10,NAT14,NAT9,SAT1,SAT2 | KAT2A,NAA10,NAA11,NAA20,NAA40,NAA50,NAGS,NNAT10,NAT14 | AANAT,KAT2A,NAA10,NAA20,NAA30,NAA40,NAA50,NAT10,NAT14,NAT9,SAT1 | GNPNAT1,NAA10,NAA11,NAA20,NAA30,NAA40,NAA50,NAT10,SAT1 | NAA20,NAA50,NAT14,NAT9,SAT1,SAT2 | NAA20,NAA50,NAT10 | AANAT,KAT2A,NAA10,NAA11,NAA20,NAA40,NAA50,SAT1 | GNPNAT1,AANAT,NAA20,NAA30,NAA40,NAA50,NAT10,SAT1,SAT2 | GNPNAT1,NAA11,NAA20,NAA50,NAT10,SAT1,SAT2 |
| LUAD | Up  regulation | NAA10,NAA20,AA40,NAA50,NAT10 | ——— | KAT2B,NAGS,NAT14,NAT16,NAT8L,SAT1,SAT2 | GNPNAT1,NAA60 | GNPNAT1,KAT2B,NAA60,SAT2 | KAT2B,NAGS,NAT8,SAT2 | NAA60,NAT14,NAT8,SAT2 | AANAT,GNPNAT1,KAT2B,NAA20,NAA30,NAA40,NAA60,NAT10 | KAT2A,SAT2 | ELP3,GNPNAT1,NAA20,NAA30,NAA60,NAT10,NAT14,NAT8L | ELP3,KAT2A,NAA11,NAA20,NAT10,NAT14 | AANAT,ELP3,KAT2A,KAT2B,NAA10,NAA40,NAT14,NAT16,NAT8L |
|  | Down  regulation | ELP3,GNPNAT1,KAT2B,NAGS,NAT14,NAT9 | ——— | GNPNAT1,KAT2A,NAA10,NA40,NAT10,NAT9 | KAT2A,NAA10,NAA20,NAA30,NAGS,NAT14,NAT9,SAT1,SAT2 | ELP3,NAA20,NAA40,NAGS,NAT14 | AANAT,ELP3,GNPNAT1,KAT2A,NAA10,NAA30,NAA40,NAT10,NAT14,NAT9,SAT1 | GNPNAT1,NAA10,NAA30,NAGS,NAT10,NAT9 | NAT14,NAT9,SAT1,SAT2 | ——— | AANAT,KAT2A,KAT2B,NAGS,NAT9,SAT1 | AANAT,GNPNAT1,NAA30,NAGS,SAT1 | GNPNAT1,NAGS,SAT1 |

**Supplemental Table 5:** Expression of general control non-repressible 5 -related N-acetyltransferases (GNAT) family members in A549 and H1299 cells 3 and 48 after CSE challenge or 6 and 48 hours after LPS challenge, respectively, measured by bulk cell RNA sequencing (Mean+SEM)

|  | A549 | | | | | | H1299 | | | | | |
| --- | --- | --- | --- | --- | --- | --- | --- | --- | --- | --- | --- | --- |
|  | Vehicle-3h | CSE-3h | LPS-6h | Vehicle-48h | CSE-48h | LPS-48h | Vehicle-3h | CSE-3h | LPS-6h | Vehicle-48h | CSE-48h | LPS-48h |
| AANAT | 0.301±0.05 | 0.147±0.039 | 0.229±0.023 | 0.123±0.027 | 0.369±0.036 | 0.239±0.021 | 0.252±0.018 | 0.099±0.013 | 0.074±0.018 | 0.071±0.007 | 0.089±0.018 | 0.119±0.043 |
| ELP3 | 11.94±0.555 | 10.06±0.418 | 10.36±0.333 | 10.51±0.123 | 10.85±0.058 | 10.62±0.043 | 6.406±0.084 | 6.275±0.095 | 4.668±0.079 | 4.503±0.017 | 7.287±0.064 | 4.542±0.185 |
| GNPNAT1 | 16.86±1.554 | 19.31±0.967 | 17.17±0.394 | 17±0.341 | 18.78±0.548 | 16.3±0.275 | 14.89±0.207 | 14.11±0.673 | 8.194±0.15 | 9.706±0.153 | 15.01±0.353 | 8.704±0.207 |
| KAT14 | 4.839±0.176 | 4.108±0.146 | 4.694±0.074 | 4.452±0.057 | 4.351±0.036 | 4.61±0.07 | 1.327±0.015 | 1.694±0.15 | 1.391±0.047 | 1.414±0.065 | 1.597±0.082 | 1.216±0.035 |
| KAT2A | 29.05±2.596 | 23.36±0.749 | 22.13±1.061 | 20.48±0.19 | 24.92±0.29 | 21.2±0.313 | 33.39±1.048 | 32.12±0.908 | 27.13±0.333 | 24.23±0.792 | 32.15±0.601 | 24.11±0.062 |
| KAT2B | 3.523±0.463 | 5.223±0.095 | 4.56±0.196 | 4.692±0.081 | 4.069±0.334 | 4.077±0.05 | 3.056±0.082 | 3.554±0.2 | 4.071±0.149 | 4.272±0.064 | 3.263±0.237 | 4.25±0.116 |
| NAA10 | 14.05±2.735 | 11.67±0.423 | 8.054±0.145 | 7.554±0.24 | 12.19±0.68 | 7.775±0.4 | 11.4±0.118 | 11.41±0.11 | 10.77±0.839 | 10.33±0.121 | 11.62±0.332 | 9.344±0.525 |
| NAA11 | 0±0 | 0±0 | 0.015±0.012 | 0±0 | 0±0 | 0±0 | 0.02±0 | 0±0 | 0±0 | 0.008±0.006 | 0.007±0.006 | 0±0 |
| NAA20 | 68.51±4.748 | 62.13±4.177 | 55.74±1.113 | 52.99±0.093 | 68.33±2.455 | 50.1±1.942 | 28.42±0.48 | 27.4±0.429 | 22.82±0.816 | 22.13±0.314 | 30.18±0.402 | 22.95±1.016 |
| NAA30 | 5.032±0.778 | 5.908±0.11 | 6.098±0.204 | 5.78±0.221 | 5.431±0.309 | 6.195±0.063 | 4.103±0.058 | 4.284±0.006 | 4.057±0.07 | 3.875±0.055 | 4.39±0.196 | 3.832±0.062 |
| NAA40 | 8.569±0.241 | 8.74±0.32 | 8.845±0.119 | 10.02±0.208 | 9.285±0.444 | 9.735±0.248 | 5.16±0.142 | 3.869±0.116 | 5.922±0.182 | 5.641±0.031 | 5.097±0.073 | 5.419±0.117 |
| NAA50 | 34.92±3.276 | 40.02±0.686 | 32.43±0.122 | 32.07±0.289 | 38.17±1.878 | 32.33±0.402 | 42.78±0.697 | 44.22±0.534 | 28.93±0.68 | 27.38±0.394 | 43.68±0.21 | 28.19±0.486 |
| NAA60 | 12±1.135 | 9.738±0.543 | 8.443±0.13 | 8.539±0.144 | 10.33±0.265 | 8.983±0.19 | 4.985±0.134 | 5.082±0.313 | 5.051±0.1 | 4.505±0.084 | 5.129±0.108 | 4.19±0.048 |
| NAA80 | 3.413±0.38 | 2.814±0.203 | 3.231±0.285 | 4.036±0.077 | 2.383±0.262 | 4.76±0.232 | 2.722±0.228 | 2.711±0.147 | 4.156±0.206 | 4.486±0.17 | 2.518±0.184 | 3.912±0.181 |
| NAGS | 0.673±0.127 | 0.48±0.057 | 0.492±0.019 | 0.537±0.031 | 0.609±0.012 | 0.607±0.158 | 1.7±0.072 | 1.326±0.076 | 3.651±0.352 | 3.438±0.179 | 2.042±0.13 | 3.376±0.17 |
| NAT10 | 16.07±0.704 | 16.28±0.159 | 12.5±0.263 | 10.66±0.069 | 16.53±0.521 | 9.953±0.159 | 14.1±0.18 | 12.57±0.325 | 7.537±0.155 | 6.964±0.175 | 13.27±0.174 | 6.891±0.119 |
| NAT14 | 29.43±3.865 | 21.78±0.292 | 27.25±1.152 | 28.41±0.897 | 21.92±0.259 | 28.71±0.845 | 14.75±0.24 | 13.91±0.676 | 25.24±1.262 | 23.86±0.149 | 14.8±0.604 | 22.75±0.58 |
| NAT16 | 0.012±0.005 | 0.011±0.004 | 0.04±0.014 | 0.08±0.009 | 0.021±0.005 | 0.16±0.016 | 0.11±0.025 | 0.157±0.018 | 0.534±0.028 | 0.591±0.015 | 0.097±0.031 | 0.642±0.062 |
| NAT8 | 0±0 | 0±0 | 0.074±0.04 | 0±0 | 0±0 | 0±0 | 0±0 | 0±0 | 0.032±0.026 | 0±0 | 0±0 | 0±0 |
| NAT8L | 8.926±0.169 | 7.912±0.369 | 8.33±0.532 | 7.635±0.248 | 7.894±0.328 | 7.941±0.118 | 20.67±0.802 | 21.11±0.682 | 16.19±0.512 | 16.38±0.27 | 17.71±0.446 | 16.52±0.555 |
| NAT9 | 10.2±1.521 | 8.802±0.49 | 7.116±0.351 | 6.493±0.278 | 8.894±0.617 | 7.02±0.33 | 11.14±0.336 | 10.12±0.441 | 8.311±0.144 | 7.972±0.053 | 10.52±0.305 | 7.888±0.232 |
| SAT1 | 141.6±0.925 | 140.6±11.46 | 334.1±2.701 | 359.9±3.686 | 156.1±3.94 | 398.9±6.955 | 18.58±0.454 | 26.36±0.911 | 22.04±1.612 | 26.79±0.355 | 19.63±0.295 | 26.92±0.452 |
| SAT2 | 14.52±2.153 | 13.03±0.654 | 14.19±0.243 | 16.93±0.861 | 13.27±0.594 | 15.71±0.247 | 14.52±0.519 | 14.48±0.419 | 19.26±1.131 | 18.26±0.251 | 15.74±0.248 | 17.62±0.439 |
| SATL1 | 0.009±0.004 | 0.003±0.002 | 0.017±0.011 | 0.009±0.008 | 0.018±0.007 | 0.022±0.008 | 0.005±0.004 | 0±0 | 0.003±0.002 | 0.006±0.002 | 0.003±0.002 | 0.007±0.003 |

Green color stands for lower expression of genes with p values less than 0.05, while yellow color for higher expression of gene with p values less than 0.05, as compared with controls, respectively.

**Supplemental Table 6:** Expression of general control non-repressible 5 -related N-acetyltransferases (GNAT) family members in human bronchial epithelia (HBE) 6 and 48 hours (h) after 6% CSE challenge or in HBE treated with cholesterol (chol) at 1 μg/ml 6 and 24 hours after 6% CSE, respectively, measured by bulk cell RNA sequencing (Mean+SEM)

|  | Control | Vehicle-6h | CSE-6h | Vehicle-48h | CSE-48h |  | Vehicle | Chol-6h | CSE-24h | Chol-24h | CSE+Chol-24h |
| --- | --- | --- | --- | --- | --- | --- | --- | --- | --- | --- | --- |
| AANAT | 0.175±0.03 | 0.138±0.012 | 0.123±0.04 | 0.097±0.019 | 0.128±0.045 | AANAT | 0.363±0.048 | 0.286±0.033 | 0.162±0.067 | 0.253±0.051 | 0.24±0.048 |
| ELP3 | 4.748±0.078 | 4.418±0.137 | 4.487±0.07 | 4.413±0.14 | 4.232±0.145 | ELP3 | 4.322±0.082 | 4.399±0.129 | 4.452±0.237 | 4.346±0.094 | 4.592±0.057 |
| GNPNAT1 | 27.78±0.988 | 25.8±0.856 | 27.49±0.852 | 20.75±0.52 | 22.34±0.679 | GNPNAT1 | 17.26±0.378 | 18.13±0.409 | 17.12±2.72 | 19.31±0.601 | 20.53±0.848 |
| KAT14 | 5.229±0.1 | 6.344±0.167 | 5.655±0.045 | 6.999±0.075 | 6.456±0.275 | KAT14 | 6.368±0.198 | 5.664±0.254 | 5.099±0.363 | 7.862±0.321 | 5.337±0.197 |
| KAT2A | 28.29±0.721 | 22.72±1.037 | 21.23±0.45 | 18.07±0.151 | 18.4±0.49 | KAT2A | 19.38±0.384 | 16.62±0.294 | 28.35±3.587 | 17.53±0.414 | 21.59±0.54 |
| KAT2B | 0.517±0.045 | 0.696±0.042 | 0.726±0.012 | 0.849±0.055 | 0.814±0.033 | KAT2B | 0.935±0.048 | 1.488±0.089 | 0.506±0.163 | 1.201±0.033 | 0.891±0.048 |
| NAA10 | 6.454±0.276 | 5.369±0.169 | 6.055±0.105 | 4.243±0.145 | 3.694±0.06 | NAA10 | 4.214±0.058 | 3.729±0.089 | 11.1±4.217 | 3.437±0.127 | 4.141±0.297 |
| NAA11 | 0±0 | 0±0 | 0±0 | 0±0 | 0±0 | NAA11 | 0±0 | 0±0 | 0±0 | 0.014±0.011 | 0±0 |
| NAA20 | 23.8±0.609 | 23.05±0.307 | 28.39±0.136 | 21.2±0.37 | 25.27±0.461 | NAA20 | 18.14±0.05 | 17.62±0.74 | 31.63±1.465 | 18.14±0.844 | 27.11±1.025 |
| NAA30 | 5.155±0.057 | 5.235±0.082 | 5.945±0.049 | 4.478±0.149 | 5.247±0.119 | NAA30 | 5.037±0.07 | 6.156±0.258 | 4.975±0.374 | 5.844±0.116 | 5.634±0.09 |
| NAA40 | 14.96±0.413 | 15.4±0.081 | 13.7±0.208 | 11.9±0.374 | 11.18±0.093 | NAA40 | 15.28±0.341 | 12.86±0.397 | 12.01±1.047 | 14.41±0.115 | 14.67±0.322 |
| NAA50 | 71.04±0.44 | 66.34±1.694 | 71.03±0.8 | 44.8±0.763 | 47.53±0.916 | NAA50 | 42.1±0.482 | 40.82±1.191 | 44.65±6.188 | 41.3±1.103 | 52.15±1.234 |
| NAA60 | 5.825±0.087 | 5.606±0.233 | 6.109±0.105 | 5.536±0.031 | 6.242±0.11 | NAA60 | 6.284±0.129 | 6.782±0.348 | 8.51±1.269 | 6.7±0.132 | 6.42±0.244 |
| NAA80 | 3.724±0.204 | 4.012±0.37 | 3.931±0.069 | 5.34±0.124 | 4.772±0.172 | NAA80 | 7.72±0.027 | 5.835±0.28 | 9.154±2.298 | 5.608±0.075 | 5.313±0.287 |
| NAGS | 0.542±0.057 | 0.846±0.179 | 0.707±0.08 | 0.866±0.12 | 0.899±0.054 | NAGS | 1.246±0.138 | 1.453±0.062 | 0.96±0.264 | 1.1±0.117 | 0.943±0.031 |
| NAT10 | 57.29±0.471 | 47.62±0.549 | 43.91±0.659 | 37.58±0.559 | 36.17±0.272 | NAT10 | 37.21±0.318 | 27.14±0.509 | 32.23±1.242 | 33.4±0.396 | 35.01±0.698 |
| NAT14 | 11.51±0.347 | 10.32±0.094 | 8.164±0.185 | 12.95±0.918 | 11.48±0.958 | NAT14 | 16.64±0.271 | 10.77±0.715 | 25.98±7.554 | 12.43±0.316 | 11.84±0.828 |
| NAT16 | 0±0 | 0.01±0.004 | 0.02±0.008 | 0.023±0.008 | 0.038±0.004 | NAT16 | 0.017±0.008 | 0.025±0.011 | 0.036±0.018 | 0.01±0.004 | 0.027±0.016 |
| NAT8 | 0.02±0.016 | 0±0 | 0±0 | 0±0 | 0±0 | NAT8 | 0±0 | 0±0 | 0±0 | 0±0 | 0±0 |
| NAT8L | 8.847±0.131 | 7.442±0.184 | 7.464±0.042 | 5.944±0.084 | 7.367±0.271 | NAT8L | 4.952±0.224 | 4.321±0.081 | 6.218±0.412 | 3.804±0.064 | 5.931±0.385 |
| NAT9 | 11.13±0.364 | 9.284±0.108 | 10.52±0.323 | 8.378±0.213 | 8.623±0.323 | NAT9 | 8.569±0.305 | 6.328±0.198 | 7.694±1.093 | 7.244±0.354 | 6.69±0.309 |
| SAT1 | 70.45±1.089 | 74.83±1.019 | 134.3±0.203 | 156.2±1.765 | 177.4±3.534 | SAT1 | 78.36±1.085 | 65.45±3.058 | 131.3±7.047 | 69.54±1.616 | 107.1±4.257 |
| SAT2 | 11±0.365 | 10.25±0.519 | 12.24±0.369 | 12.5±0.783 | 13.73±0.745 | SAT2 | 10.82±0.56 | 12.76±0.643 | 25.4±7.132 | 11.69±0.325 | 12.2±0.373 |
| SATL1 | 0±0 | 0±0 | 0±0 | 0±0 | 0±0 | SATL1 | 0±0 | 0.008±0.007 | 0.003±0.002 | 0±0 | 0.006±0.005 |

Green color stands for lower expression of genes with p values less than 0.05, while yellow color for higher expression of gene with p values less than 0.05, as compared with controls, respectively.

**Supplemental Table 7:** Expression of general control non-repressible 5 -related N-acetyltransferases (GNAT) family members in human SPC-16 and 24 hours (h) after LPC challenge or treated H460 with AR2 inhibitor (306) at 0,0.5,2.5,5.0nM for 24 hours, respectively, measured by bulk cell RNA sequencing (Mean+SEM)

|  | SPC-1 | | | |  | H460 | | | |
| --- | --- | --- | --- | --- | --- | --- | --- | --- | --- |
|  | Vehicle-6h | LPC-100μl-6h | Vehicle-24h | LPC-100μl-24h |  | Control | H460+306(0.5) | H460+306(2.5) | H460+306(5.0) |
| AANAT | 0.145±0.061 | 0.11±0.017 | 0.084±0.022 | 0.059±0.006 | AANAT | 0.626±0.088 | 0.725±0.053 | 0.581±0.054 | 0.375±0.005 |
| ELP3 | 9.209±0.205 | 9.094±0.204 | 8.728±0.246 | 9.393±0.15 | ELP3 | 8.467±0.052 | 8.544±0.126 | 8.649±0.157 | 11.19±0.205 |
| GNPNAT1 | 10.13±0.539 | 8.278±0.484 | 8.586±0.405 | 7.727±0.375 | GNPNAT1 | 10.21±0.123 | 11.04±0.38 | 9.954±0.151 | 10.43±0.231 |
| KAT14 | 1.191±0.143 | 1.15±0.104 | 1.433±0.052 | 1.277±0.062 | KAT14 | 2.74±0.024 | 2.962±0.1 | 2.773±0.072 | 2.029±0.077 |
| KAT2A | 31.75±0.684 | 29.65±1.2 | 27.2±0.57 | 32.13±0.619 | KAT2A | 33.67±0.708 | 35.49±1.026 | 33.95±1.158 | 24.35±0.886 |
| KAT2B | 1.019±0.022 | 1.56±0.095 | 1.199±0.089 | 1.76±0.079 | KAT2B | 2.723±0.137 | 2.723±0.111 | 2.861±0.068 | 2.03±0.138 |
| NAA10 | 12.21±1.688 | 6.794±0.321 | 8.024±0.919 | 6.519±0.02 | NAA10 | 10.94±0.213 | 11.71±0.502 | 10.84±0.477 | 12.92±0.544 |
| NAA11 | 0±0 | 0±0 | 0±0 | 0±0 | NAA11 | 0.01±0.008 | 0±0 | 0±0 | 0±0 |
| NAA20 | 39.68±1.063 | 21.03±0.906 | 33.77±2.079 | 21.45±1.105 | NAA20 | 41.52±0.216 | 42.04±0.74 | 35.46±0.631 | 32.54±0.287 |
| NAA30 | 1.933±0.098 | 2.261±0.262 | 2.11±0.129 | 2.247±0.024 | NAA30 | 7.121±0.111 | 6.957±0.135 | 6.606±0.086 | 8.154±0.201 |
| NAA40 | 3.619±0.096 | 4.719±0.351 | 3.431±0.076 | 4.799±0.117 | NAA40 | 11.74±0.163 | 11.58±0.319 | 8.772±0.347 | 6.612±0.233 |
| NAA50 | 39.49±3.199 | 34.59±1.28 | 25.79±1.25 | 34.43±0.556 | NAA50 | 21.66±0.453 | 21.96±1.026 | 22.48±0.563 | 22.05±0.279 |
| NAA60 | 6.335±0.567 | 8.439±0.532 | 6.358±0.171 | 8.305±0.238 | NAA60 | 15.03±0.261 | 16.27±0.549 | 15.24±0.768 | 20.21±0.84 |
| NAA80 | 7.026±0.588 | 10.84±0.146 | 10.14±0.923 | 9.685±0.23 | NAA80 | 0±0 | 0±0 | 0±0 | 0±0 |
| NAGS | 0.857±0.154 | 1.036±0.023 | 1.188±0.146 | 0.616±0.023 | NAGS | 1.002±0.012 | 1.259±0.06 | 0.936±0.02 | 1.213±0.153 |
| NAT10 | 25.31±3.004 | 26.94±2.006 | 18.01±0.367 | 24.4±0.333 | NAT10 | 24.07±0.441 | 24.11±0.46 | 20.51±0.171 | 17.96±0.194 |
| NAT14 | 26.03±2.951 | 18.9±1.001 | 29.42±2.77 | 17.33±0.445 | NAT14 | 22.61±1.107 | 24.25±1.109 | 17.1±0.835 | 13.7±0.218 |
| NAT16 | 0.012±0.005 | 0.027±0.013 | 0.027±0.009 | 0.021±0.012 | NAT16 | 0.044±0.008 | 0.039±0.003 | 0.088±0.024 | 0.042±0.003 |
| NAT8 | 0±0 | 0±0 | 0±0 | 0±0 | NAT8 | 0.029±0.024 | 0±0 | 0.048±0.023 | 0±0 |
| NAT8L | 1.647±0.134 | 2.71±0.134 | 1.48±0.072 | 1.858±0.076 | NAT8L | 7.621±0.248 | 8.369±0.482 | 5.965±0.19 | 4.694±0.069 |
| NAT9 | 8.418±0.46 | 7.889±0.387 | 7.692±0.543 | 5.752±0.12 | NAT9 | 14.31±0.273 | 14.79±0.586 | 15.4±0.777 | 20.43±0.699 |
| SAT1 | 15.12±1.453 | 14.49±1.46 | 15.67±0.284 | 14.15±0.336 | SAT1 | 95.62±1.353 | 92.18±2.052 | 116.2±2.831 | 114.9±2.466 |
| SAT2 | 29.15±2.417 | 18.23±0.585 | 29.95±2.684 | 18.82±0.141 | SAT2 | 9.207±0.254 | 8.223±0.23 | 10.29±0.559 | 16.22±1.196 |
| SATL1 | 0.01±0.005 | 0±0 | 0±0 | 0±0 | SATL1 | 0±0 | 0±0 | 0.054±0.036 | 0.026±0.022 |

Green color stands for lower expression of genes with p values less than 0.05, while yellow color for higher expression of gene with p values less than 0.05, as compared with controls, respectively.
