# Supplementary figures and images for: Transcriptomic atlas of GNAT family members in pulmonary epithelia under pathological conditions using single‐cell and bulk cell sequencing
Source: Clin Transl Med. 2022 Jul 20;12(7):e841. doi: 10.1002/ctm2.841 (PMC9299758; doi:10.1002/ctm2.841)

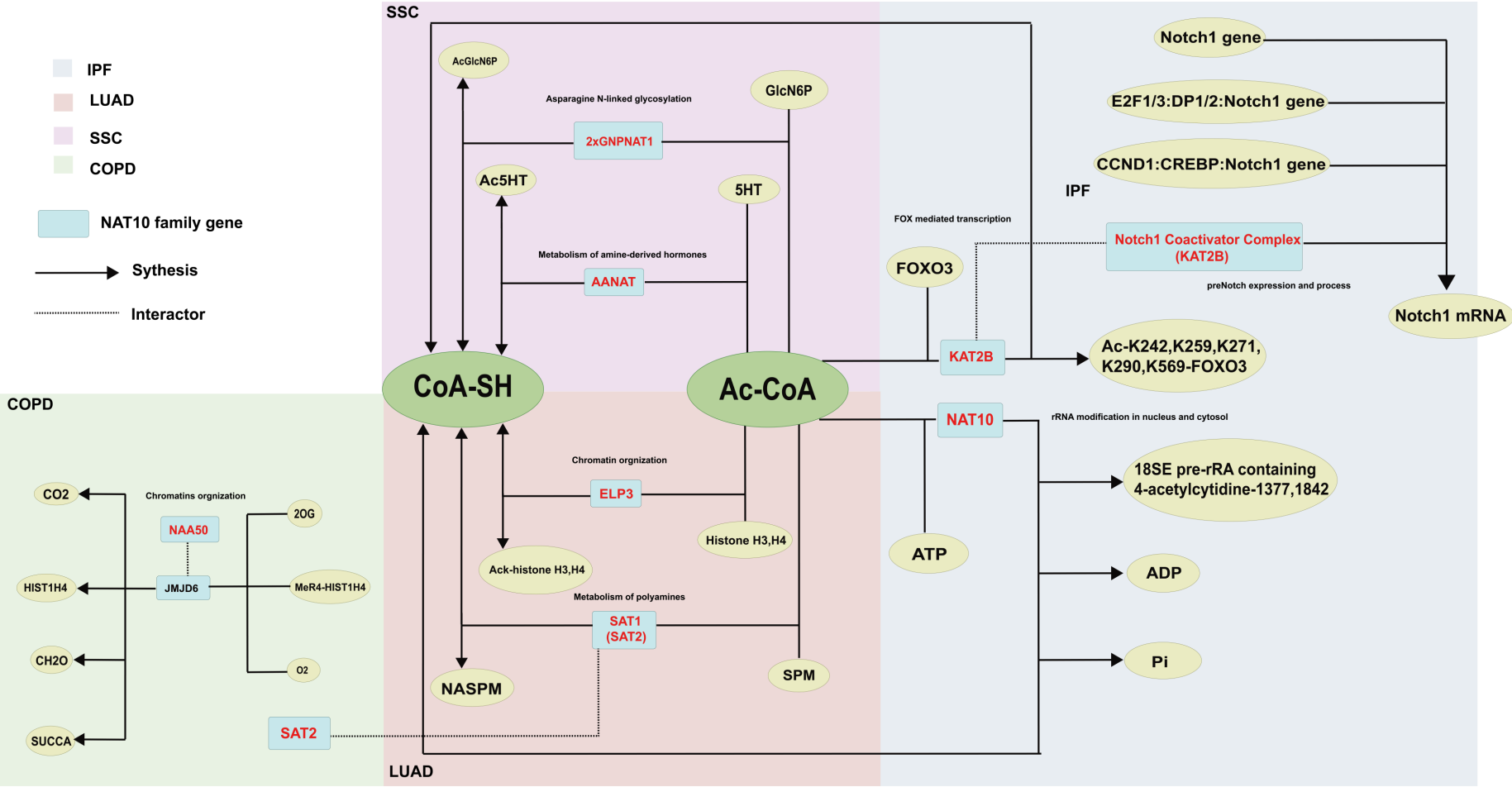

Supplemental Figure 1

Supplement: Supplementary file 2 — Supplementary material [file CTM2-12-e841-s002.pdf]
