# Supplementary material for: Transcriptomic atlas of GNAT family members in pulmonary epithelia under pathological conditions using single‐cell and bulk cell sequencing
Source: Clin Transl Med. 2022 Jul 20;12(7):e841. doi: 10.1002/ctm2.841 (PMC9299758; doi:10.1002/ctm2.841)
Supplement: Supplementary file 3 — Supplementary material [file CTM2-12-e841-s001.docx]

**Supplement Methods:**

**GNAT family gene in Lung single cell sequence analysis:**

The scRNAseq data was analyzed including 84 lung tissue samples from 20 healthy controls, 15 chronic obstructive pulmonary disease (COPD) patients, 15 idiopathic pulmonary fibrosis (IPF) patients, 8 SSC patients, 15 lung adenocarcinoma (LUAD) patients and 11 corresponding para cancer tissues. All scRNA-seq data can be accessed at GEO database (<https://www.ncbi.nlm.nih.gov/geo/>) and ArrayExpress, accession number: GSE128169 (Valenzi et al, 2019), GSE131907_Lung_Cancer (Kim et al, 2020), GSE136831 (Adams et al, 2020), GSE128033 (Morse et al, 2019), E-MTAB-6653 and E-MTAB-6149 (Lambrechts et al, 2018). We transformed the raw data into gene expression matrices using cell ranger 706 3.0.0 (10x Genomics), and constructed Seurat object using the Seurat R package (version 3.0). The datasets were annotated with Seurat R and unsupervised clustering of cells obtained from the identified 55 clusters. After the standardization of gene expression profile, we further detected the relative expression of GNAT family gene members in the cluster of 8 epithelial cells isolated from 5 kinds lung tissues. The cell labels of epithelial cells were as listed: HOPX, PDPN, CLIC5, AGER, and CLDN18 for alveolar epithelial type 1; SFTPB, SFTPC, SFTPD, ETV5, and MUC1 for alveolar epithelial type 2; SCGB1A1 and SCGB3A2 for club epithelia; FOXJ1 for ciliated epithelia; KRT5 and KRT14 for basal cells; MUC5AC and MUC5B for goblet cells/mucus epithelial cells; CALCA, CHGA and ASCL1 for neuroendocrine.

**GNAT family gene in pan cancer analysis:**

The GNAT family gene expression data of TCGA pan cancer and Genotype-Tissue Expression ( GTEx) from the University of California Santa Cruz(UCSC) Xena browser (https://xena.ucsc.edu/), (https://portals.broadinstitute.org/ccle/data),(<https://gtexportal.org/home/datasets>). A total of 15 kinds of tissues were finally obtained which include normal lung tissues (n=574), lung adenocarcinoma(n=397), Para-adenocarcinoma lung tissue(n=59), Lung squamous carcinoma(n=285), Para-squamous carcinoma lung tissue(n=50), Glioblastoma multiforme(n=153), Bladder Urothelial Carcinoma(n=1092), Breast invasive carcinoma(n=285), Cholangiocarcinoma(n=36), Esophageal carcinoma(n=181), Kidney renal papillary cell carcinoma(n=369), Liver hepatocellular carcinoma(n=495), Prostate adenocarcinoma(n=414), Stomach adenocarcinoma(n=504), Thyroid carcinoma(n=513). The count value expression matrix was extracted first . In order to further acquire the FPKM value of GNAT family genes, we download gff3( <http://ftp.ebi.ac.uk/pub/databases/gencode/Gencode_human/release_22/gencode.v22.annotation.gff3.gz>) from GENCODE (<https://www.gencodegenes.org/human/>). From this data, we extracted the unique 3'UTR length for each gene. And then the value of FPKM of each gene was calculated according to the gene length and the mapped reads count. The transformation formula of FPKM value was as follows: FPKM= total exon reads/ (mapped reads (Millions) * exon length (KB)). The results shown are representative of gene expression and the mean values ± standard error of the mean (SEM) from various samples. Statistical evaluations were carried out with Prism 9.0 (GraphPad Software, Inc.). One-way ANOVA with Dunnett’s test was used for comparing multiple tissues to the LUAD tissue or LUSC tissue. P values < 0.05 were considered statistically significant.

**GNAT family gene in bulk RNA sequencing and analysis**

Cellular samples were collected in TRIZOL and RNA quality was assessed by RNA Nano 6000 Assay Kit of the Bioanalyzer 2100 system. The amount of 1 μg RNA per sample was used as RNA sample preparations. And then mRNA was purified from total RNA using poly-T oligo-attached magnetic beads. Fragmentation was carried out using divalent cations under elevated temperature in First Strand Synthesis Reaction Buffer (5X). First strand: cDNA was synthesized using random hexamer primer and M-MuLV Reverse Transcriptase. Second strand: cDNA synthesis was subsequently performed using DNA Polymerase I and RNase H. The rest overhangs were converted into blunt ends via exonuclease/polymerase activities. After adenylation of 3’ ends of DNA fragments, Adaptor with hairpin loop structure were ligated to prepare for hybridization. The library fragments were purified with AMPure XP system (Beckman Coulter, Beverly, USA) to select cDNA fragments of preferentially 370~420 bp in length. PCR was performed using Phusion High-Fidelity DNA polymerase, Universal PCR primers, and Index (X) Primer.And then PCR products were purified (AMPure XP system) and library quality was assessed (Agilent Bioanalyzer 2100). The clustering of the index-coded samples was performed according to the manufacturer’s protocol. After cluster generation, prepared libraries were sequenced on Illumina Novaseq to generate 150bp paired-end reads. After quality control of sequencing data, the index of the reference genome was built and paired-end clean reads were aligned to the reference genome using Hisat2 v2.0.5. Read numbers were counted and mapped to each gene via FeatureCounts v1.5.0-p3. According to length of the gene and reads count mapped to the gene, the fragments per kilobase of transcript per million mapped reads of each gene were calculated. Bulk RNA-seq analysis were performed based on the samples with per group were biological triplicates. Differentially expressed genes were assessed using the One-way ANOVA test in Graph pad Prism 9.0 for comparing multiple conditions to the control group. Genes with p-values<0.05 were selected as significantly changed among various conditions.
